# Effect of Surface and Bulk Properties of Mesoporous Carbons on the Electrochemical Behavior of GOx-Nanocomposites

**DOI:** 10.3389/fchem.2019.00084

**Published:** 2019-02-19

**Authors:** Tsai Garcia-Perez, Shouzhen Hu, Youngho Wee, Louis Scudiero, Conrad Hoffstater, Jungbae Kim, Su Ha

**Affiliations:** ^1^School of Chemical Engineering and Bioengineering, Washington State University, Pullman, WA, United States; ^2^Department of Chemical and Biological Engineering, Korea University, Seoul, South Korea; ^3^Department of Chemistry and Materials Science and Engineering Program, Washington State University, Pullman, WA, United States

**Keywords:** graphitized mesoporous carbon, graphitization index, hydrophobic properties, biofuel cells, glucose oxidase, enzymatic nanocomposites

## Abstract

Biofuel cell (BFC) electrodes are typically manufactured by combining enzymes that act as catalysts with conductive carbon nanomaterials in a form of enzyme-nanocomposite. However, a little attention has been paid to effects of the carbon nanomaterials' structural properties on the electrochemical performances of the enzyme-nanocomposites. This work aims at studying the effects of surface and bulk properties of carbon nanomaterials with different degrees of graphitization on the electrochemical performances of glucose oxidase (GOx)-nanocomposites produced by immobilizing GOx within a network of carbon nanopaticles. Two types of carbon nanomaterials were used: graphitized mesoporous carbon (GMC) and purified mesoporous carbon (PMC). Graphitization index, surface functional groups, hydrophobic properties, and rate of aggregation were measured for as-received and acid-treated GMC and PMC samples by using Raman spectrometry, *X-ray photoelectron spectroscopy (*XPS), contact angle measurement, and dynamic light scattering (DLS), respectively. In addition to these physical property characterizations, the enzyme loading and electrochemical performances of the GOx-nanocomposites were studied via elemental analysis and cyclic voltammetry tests, respectively. We also fabricated BFCs using our GOx-nanocomposite materials as the enzyme anodes, and tested their performances by obtaining current-voltage (IV) plots. Our findings suggest that the electrochemical performance of GOx-nanocomposite material is determined by the combined effects of graphitization index, electrical conductivity and surface chemistry of carbon nanomaterials.

## Introduction

Self-powered implantable devices such as deep brain neurostimulators, pacemakers, and biosensors for environmental monitoring have enormous potential in medical, agricultural or even military applications (Falk et al., 2012; Katz, 2013). Biofuel cells (BFCs) can be an alternative portable power solution to batteries for powering these devices, due to their capability to continuously convert the chemical energy from organic fuels, such as glucose in fruits or human blood, into electricity (Katz, 2013; MacVittie et al., 2015). Enzymatic BFCs use (1) enzymes to catalyze both oxidation of organic fuels and reduction of oxidizing agents, and (2) conductive materials (such as carbon nanomaterials) to transmit the electrons between the enzymes' active sites and the electrodes. Thus, the physical properties of both materials—enzymes and nanomaterials—play a key role in the BFC's electrochemical performances. However, to the best of our knowledge, little attention has been paid to the effects of the carbon nanoparticle's surface and bulk properties on the overall electrochemical performance of the enzyme electrodes.

Graphitized mesoporous carbons (GMC) and purified mesoporous carbons (PMC) are two types of mesoporous carbon materials with similar chemical composition and morphological properties, but different surface and structural properties. This contrast on the properties of GMC and PMC makes them the ideal carbon nanomaterials for investigating the effects of carbon nanomaterials' properties on the electrochemical performances of BFCs.

Graphitized and non-graphitized carbons are structurally different (Franklin, 1951). The graphitization process of the carbon is a method to produce well-organized graphite (Mattia et al., 2006). Non-graphitized carbons exhibit a cross-linked structure where graphitic structures are randomly oriented in a rigid mass. Conversely, graphitized carbons present a compact structure where the graphite layers have a nearly parallel orientation. Graphite layers play a major role in both the surface and bulk properties of these materials. The hydrophobicity and electrical conductivity of the carbon materials, for example, are directly related to the level of graphitization of the carbon materials (Pantea et al., 2003). Hydrophobicity also affects the nanoparticle aggregation process (Nel et al., 2009). It is known that the surface of graphitized materials presents smaller amounts of oxygen functionalities compared to that of non-graphitized materials, which strongly repels water molecules (due to their hydrophobic nature) and decreases electrostatic repulsion among the nanoparticles. Consequently, these nanomaterials can form a compact carbon network with low dispersion in an aqueous medium. These unique properties of nanomaterials have been used to physically entrap large enzyme aggregates within the carbon networks and to form protein-nanocomposite materials (Garcia-Perez et al., 2016). This observation suggests that the performance of this hybrid protein-nanoparticle composite structure highly depends on the graphitization index of the carbon nanomaterial used as the enzyme support. Literature shows that the GMC sample can be used to entrap enzymes to build bioelectrodes (Garcia-Perez et al., 2016; Walcarius, 2017), although no information has been reported in the literature on employing the PMC sample for electrochemical applications.

This work aims to study the effects of the bulk and the surface properties of different carbon nanomaterials on the electrochemical performances of glucose oxidase (GOx)-nanocomposite bioanode materials under the BFC operation mode. For the present study, a homemade Proton Exchange Membrane (PEM) fuel cell was used to test the enzymatic BFC performances. Four different bioanodes were manufactured using GOx as catalysts and (a) GMC, (b) PMC, (c) acid treated GMC, and (d) acid treated PMC as carbon supports.

## Materials and Methods

### Materials

Graphitized mesoporous carbons (GMC) (specific surface area of 70 m^2^/g and average pore diameter of 13.7 nm, purity >99.95%) and purified mesoporous carbons (PMC) (specific surface area of 200 m^2^/g and average pore diameter of 6.4 nm, purity >99.95%) were purchased from Sigma Aldrich. Glucose oxidase (GOx) Type VII from *Aspergillus niger*, glutaraldehyde (8% in water), ammonium sulfate (molecular biology grade), Nafion® (5 wt% in a mixture of water and lower aliphatic alcohols), D-glucose, o-Dianisidine dihydrochloride, and Peroxidase Type II from *Horseradish* were also obtained from Sigma Aldrich. Nanopure water (>18 MΩ-cm) was used to prepare sodium phosphate buffer (hereafter referred to as PB) and Tris-buffer solutions. The glucose solutions used during the electrochemical tests were prepared 1 day in advance to allow mutarotation of α-glucose to β-glucose.

### Methods

#### GOx-Nanocomposites Preparation

The GOx-nanocomposites were prepared using cross-linked GOx aggregates as the catalyst and the GMC or the PMC as the carbon supports. The carbon materials were first acid treated using 25 ml of a mixture of nitric acid and sulfuric acid in a volume ratio of 1:3. The acids were mixed in a 50 ml bottle and allowed to cool down to room temperature. Then, 0.25 g of the carbon nanoparticles were added into the acid mixture, as they were stirred with a magnetic bar. The suspension was then shaken for 24 h. Following the acid treatment, the samples were washed with nanopure water and vacuum filtered. The GOx-nanocomposites were prepared by adding 5 ml of ammonium sulfate solution (0.5 mg/ml) to 2.5 ml of GOx solution (1 mg of GOx/ml) to precipitate the enzymes. Then, 0.13 vol% of glutaraldehyde was added to the solution in order to obtain the cross-linked GOx aggregates. Next, 5 ml of nanocarbon solutions (1 mg/ml) was added and stirred for 30 min using a shaker (Max Q 2,000) to form the GOx-nanocomposites. These nanocarbon solutions included the GMC, PMC, acid treated GMC (herein referred to as GMCac), and acid treated PMC (herein referred to as PMCac) dispersed into the buffer solution. The nanocomposites were washed 6 times: 2 times with Tris-buffer solution (pH 7.2) to cap the underreacted glutaraldehyde and 4 times with sodium phosphate buffer (PB, 100 mM, pH 7.0) to remove the free GOx. Each washing process consisted of adding 10 ml of buffer to each suspension and then vortexing, shaking, and centrifuging. The supernatant was then removed. Finally, the PB solution was added to the GOx-nanocomposites to reach a final volume of 5 ml. The nanocomposite suspension in PB was stored at 4°C. The prepared samples will be referred to as: the GOx-GMC for cross-linked GOx aggregates-GMC; the GOx-GMCac for cross-linked GOx aggregates-GMC acid treated; the GOx-PMC for cross-linked GOx aggregates-PM; and the GOx-PMCa for cross-linked GOx aggregates-PMCac.

##### Elemental analysis and surface characterization

The ratio of enzyme to carbon material (mg of enzyme/mg of mesoporous carbon) present in each nanocomposite was estimated by determining the elemental composition (C, H, N) of the GOx-nanocomposites, GOx and carbon nanomaterials. The tests were conducted by Intertek Pharmaceutical Services (Intertek.com). For the elemental analysis, the GOx-nanocomposites samples were washed with PB only, skipping the Tris-buffer washing step for the elemental analysis tests to guarantee the enzymes are the only major source of nitrogen (Kim et al., 2011). Before sending the GOx-nanocomposite samples to Intertek Pharmaceutical Services, the samples were freeze-dried to remove water from the system. Both the GOx enzyme and nanomaterial samples were sent to the company for the elemental analysis without any special treatments. The residual water contents of the carbon nanomaterial samples were determined (prior to the elemental analysis) through thermogravimetric analysis (TGA) (García et al., 2013). Based on our TGA analysis, the water contents for the nanomaterial samples were negligible (i.e., there was no water content for both the GMC and GMCac samples, while the PMC and PMCac samples contained 2.4 and 5.9 weight% of water content, respectively).

The elemental composition of the GOx-nanocomposites, GOx, and the carbon nanomaterials was measured and reported in terms of weight%. The GMC, GMCac, and PMC contained no nitrogen. Unlike the GMC, GMCac, and PMC, the elemental analysis of the PMCac showed a trace of nitrogen element. However, its amount was insignificant and we can ignore its contribution for estimating the GOx amount presented in the GOx-PMCac nanocomposite sample. Since every GOx contains a fixed number of nitrogen element and GOx is the only major source of nitrogen element, we can easily approximate the total amount of GOx presented in each GOx-nanocomposite sample using the nitrogen weight% information obtained from the elemental analysis. Based on the carbon weight% information, we can also estimate the total amount of carbon nanomaterials presented in each GOx-nanocomposite sample. Based on this total amount information of both GOx and carbon nanomaterials, we can determine the ratio of enzyme to carbon nanomaterial (weight% of enzyme/weight% of carbon nanomaterial) for each GOx-nanocomposite sample.

The aggregation process of the carbon nanoparticles was studied by conducting dynamic light scattering (DLS) measurements. The size of the carbon nanoparticles aggregates was determined every 1 min. In addition, the interaction between the carbon nanomaterials and water was visualized by creating a thin film of each carbon nanomaterial and placing 20 μl of nanopure water on the top of it.

The graphitization index of the samples was determined via Raman Spectrometry. The Raman tests were carried out using a Jobin–Yvon Horiba LabRAM-HR spectrometer at 532 nm excitation wavelength. Prior to the tests, the samples were diluted 10 times in KBr. Each sample was scanned at least 6 times. All Raman measurements were conducted at room temperature. In addition, the functional groups on the surfaces of the samples were studied by means of X-ray photoelectron spectroscopy (XPS). The XPS spectra were recorded on a Kratos AXIS-165 XPS spectrometer using a monochromatized AlKα X-ray anode (1,486.6 eV) in an ultra-high vacuum system. The spectrometer was calibrated against both the Au 4f_7/2_ peak at 84.0 eV and the Ag 3d_5/2_ peak at 368.3 eV. Survey scans were recorded at 80 eV pass energy with a step size of 1 eV. C 1s core level spectra were recorded at 40 eV pass energy with a step size of 0.1 eV. CasaXPS software was used to analyze the XPS spectra of C 1s for all samples. The full width at half maximum (FWHM) were set to 0.8 eV for C-C bond, 1.2 eV for C-OH bond, 1.5 eV for C = O, O-C = O and carbonate bonds, and 2 eV for the π → π^*^ transition. Finally, all the spectra curves were smoothed using the Sawitzki-Golay algorithm with a kernel of five points.

##### Carbon nanoparticle's morphological characterization

Microscopy techniques were performed to visualize the structure of the carbon nanoparticles and the GOx-nanocomposites. A Zeiss 510 Confocal Microscope was used to conduct confocal analysis. In addition to confocal analysis, transmission electron microscopy (TEM) was carried out to visualize the distribution of the enzymes within the carbon nanoparticle network at a smaller scale. The TEM tests were conducted using a FEI TEM T20 microscopy at least in 5 different spots. The preparation of the samples was conducted using a similar step reported in a previous work (Garcia-Perez et al., 2016).

##### Electrochemical properties

Cyclic voltammetry (CV) was conducted using a conventional three electrode setup. Pt mesh and Ag/AgCl (KCl saturated) electrode were used as a counter electrode and a reference electrode, respectively. In addition, the working electrodes were prepared by placing 10 μl of the GOx-nanocomposites suspension (~1.5 mg of GOx-nanocomposite/ml of suspension) on a glassy carbon electrode using Nafion® binder (0.5% of the total volume). All of the CV tests were carried out using N_2_-saturated PB (100 mM, pH of 7.0) at room temperature, while the working electrode was rotated at 500 rpm. The N_2_-saturated PB solutions were prepared by bubbling high purity N_2_ into the solutions for 30 min before the test, followed by blanketing the solutions with N_2_ during the tests.

The enzyme anodes were prepared by physically absorbing the GOx-nanocomposites onto carbon paper disk with a geometric area of 0.332 cm^2^. A concentrated solution of GOx-nanocomposites was prepared by centrifuging an aliquot of 0.6 ml of GOx-nanocomposite from the main stoke for 5 min, removing 410 μl of supernatant, and adding 10 μl of 5% Nafion® solution into the remaining pellet. After that, the carbon disks were added one by one to the suspension and shaken for 10 min. The electrodes were then removed and dried for 1 h under hood conditions. Finally, the electrodes were washed three times with 100 mM PB buffer (pH 7.0) and stored at 4°C before use.

The electrochemical properties of the GMC, GMCac, PMC, and PMCac samples were studied by using CV tests. The electrodes were prepared by placing 40 μl of a nanoparticle suspension (1 mg of carbon nanoparticle/ml of ethanol) on the glassy carbon electrode and let it dry at room temperature. The ferrocyanide/ferricyanide couple is a benchmark used in our electrochemical measurements to determine the electron transfer characteristics of each electrode. The CV plots were obtained in the presence of 1 mM of potassium ferricyanide in PB (100 mM, pH 7.0) at scan rate of 10 mV s^−1^. The current density (μA/cm^2^) was determined by dividing the current obtained in the CV test with the total surface area of carbon nanomaterial (see Materials).

A homemade PEM fuel cell was used to evaluate the electrochemical performance of the GOx-nanocomposites under the enzymatic biofuel cell (BFC) operating mode as presented in previous papers (Fischback et al., 2006; Garcia-Perez et al., 2016).

## Results and Discussion

### Morphology, Graphitization Index, Surface Chemistry, and Wettability of GMC and PMC

**Figure 1:**
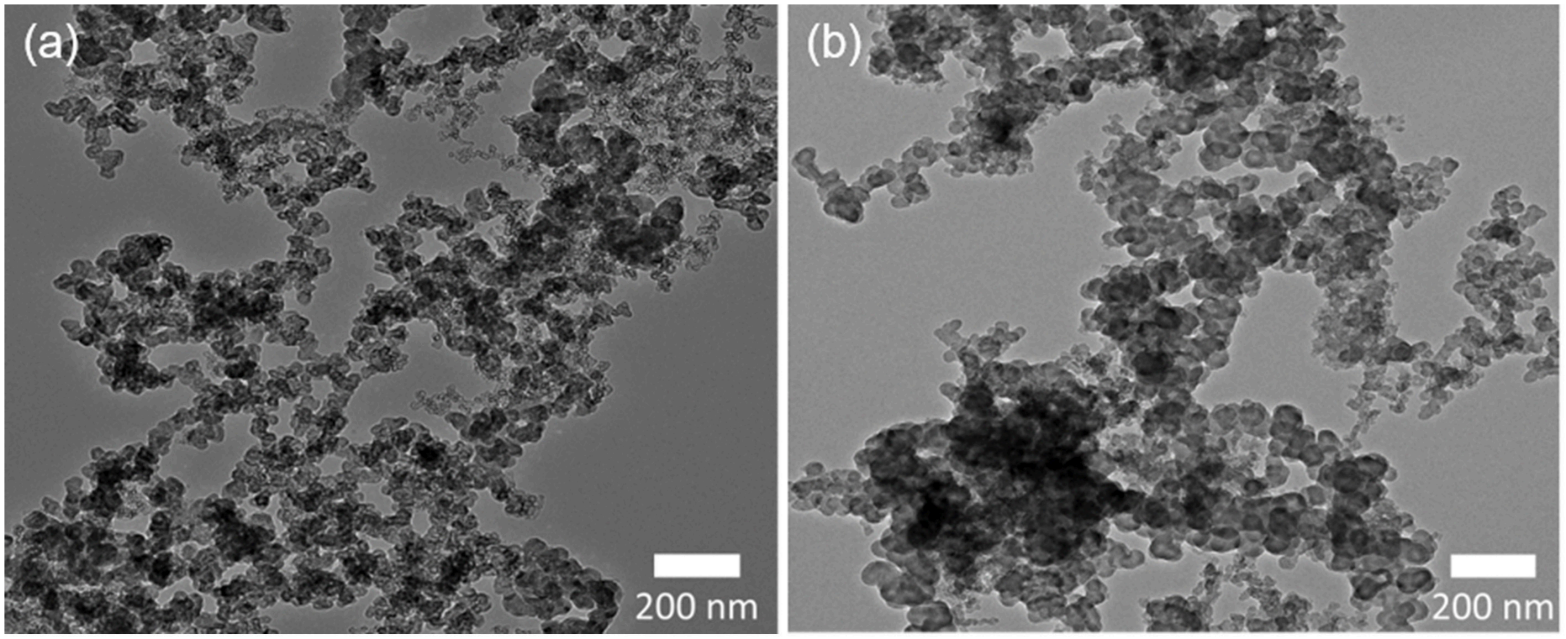
TEM images of as-received carbon nanomaterials: **(a)** GMC and **(b)** PMC.

**Figure 2:**
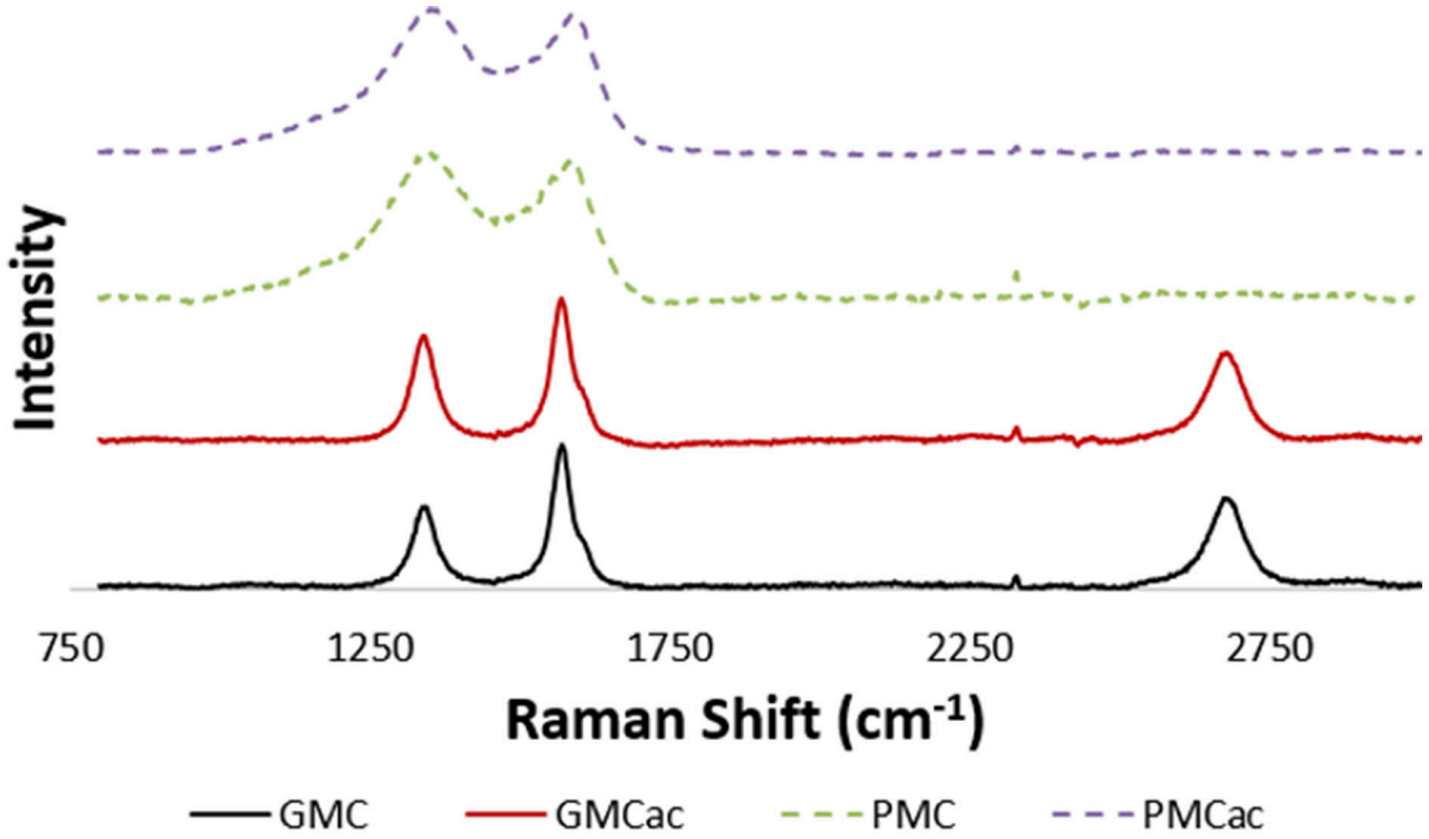
Raman spectra of GMC, GMCac, PMC, and PMCac.

Figure 1 presents the TEM images of as-received GMC and as-received PMC. Both carbon nanomaterials present similar polygonal conformations and dimensions in the nanoscale range, but they offer different specific surface areas (200 m^2^/g for PMC and 70 m^2^/g for GMC) according to their vendor information. Figure 2 presents the Raman spectra of the four carbon nanomaterials used in this work (GMC, PMC, GMCac, and PMCac). The GMC sample presents large G and D peaks at around 1,582 cm^−1^ and 1,350 cm^−1^, respectively. The GMC sample also shows a strong D′ peak at around 2,700 cm^−1^. These sharp G peak and strong G′ peak shown in the Raman spectrum of the GMC sample indicate that the GMC is consisted of a multilayer of graphene and, consequently, it presents a very organized bulk structure. Furthermore, the *full width half maximum* (FWHM) of the G and D peaks for the GMC and PMC samples show different values: around 39–45 cm^−1^ (G-D peak) for the GMC and 111–206 cm^−1^ (G-D peak) for the PMC (see Table 1). The higher FWHM values for the PMC's G and D peaks as well as the disappearance of its G′ peak at around 2,700 cm^−1^ indicate a lack of three-dimensional order of PMC materials, which is probably due to its turbostratic conformation (Ferrari, 2007). In summary, the GMC sample presents a highly graphitized structure, while the PMC sample mainly consists of turbostratic carbon structures with a low degree of bulk organization.

**Table 1:** A summary of Raman spectra for the GMC, GMCac, PMC and PMCac samples.

**Samples**	**FWHM (G band)**	**FWHM (D band)**	***I_***G***_/I_***D***_***	***1/(I_***G***_/I_***D***_)***
GMC	39.05	45.95	1.87	0.54
GMCac	44.73	47.91	1.45	0.69
PMC	111.24	206.22	–	–
PMCac	112.73	212.74	–	–

The graphitization index was calculated as an intensity ratio between the G peak and the D peak (*IG/ID*) of Raman spectra, and their inverse values (*ID/IG*) were used to quantify the defects in the carbon structure (Table 1). The high graphitization index of the as-received GMC (1.87) and GMCac (1.45) confirms that these materials present a well-organized bulk structure. However, the graphitization index of GMCac is lower than that of the as-received GMC (a reduction of ~22%), suggesting that the acid treatment creates defects on the GMC. For amorphous materials, this G peak can be attributed to the presence of benzene rings that are condensed into the amorphous structure (Schwan et al., 1996). Therefore, the intensity of this G peak (*I*_*G*_) and *IG/ID* peak ratios cannot be used to quantify the graphitization index of PMC materials. Thus, both the *IG/ID* and *ID/IG* peak ratios of both the PMC and PMCac samples are not included in Table 1.

**Figure 3:**
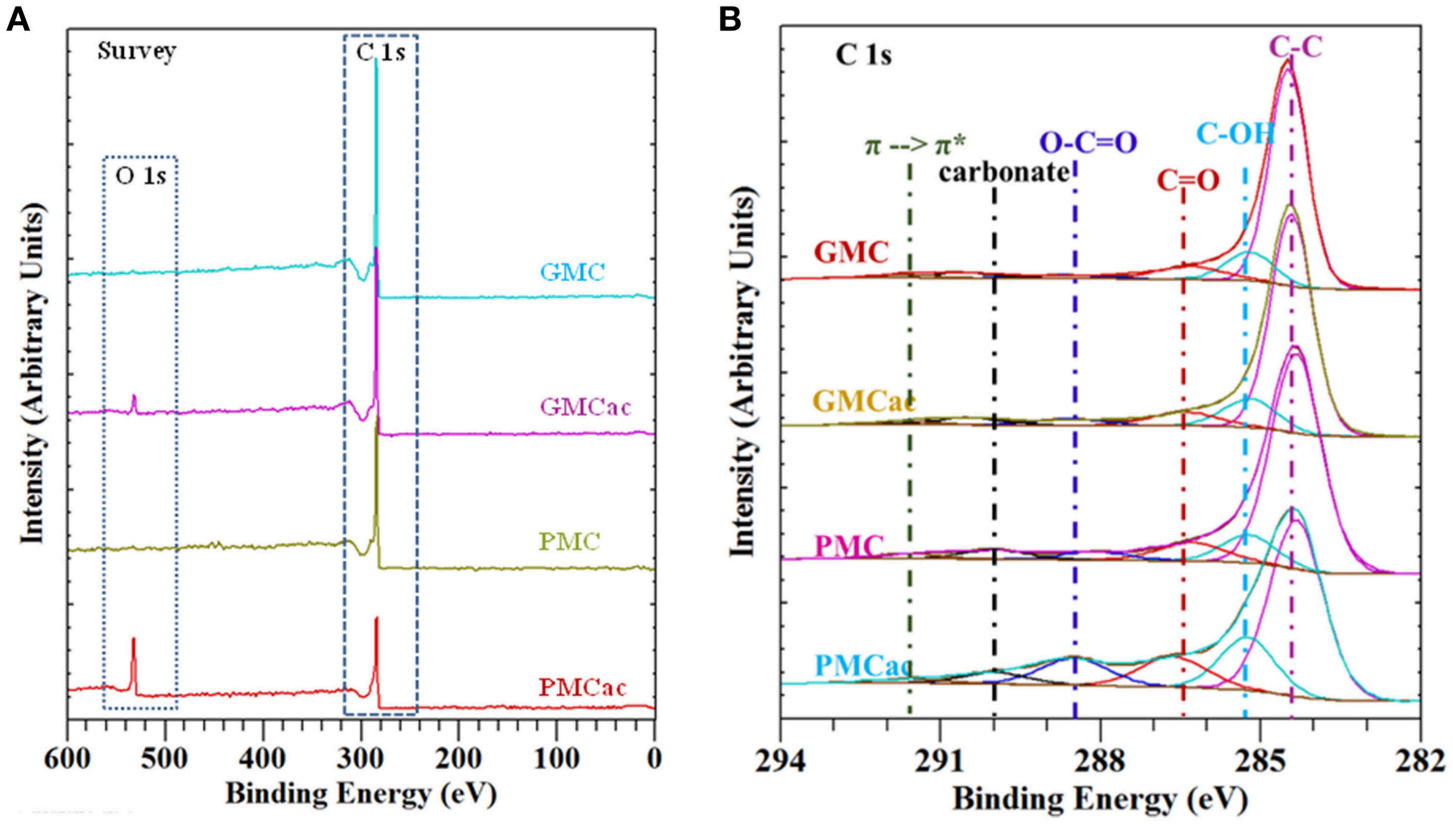
**(A)** XPS survey spectra and **(B)** curve fitted XPS C 1s peaks for GMC, GMCac, PMC, and PMCac.

The functional groups present on the surface of the GMC, GMCac, PMC, and PMCac nanomaterials were determined by XPS measurements. The XPS spectra are presented in Figure 3. As shown in Figure 3A, a main peak at around 284 eV for C 1s in the XPS survey scan was detected for all four samples. New peaks around 532 eV are observed in the XPS survey scans for both the GMCac and the PMCac samples. Such new peaks were identified as O 1s, which indicate that the surface of GMC and PMC nanomaterials are functionalized after the acid treatment. The effect of the acid treatment can be analyzed from the ratio between the amount of O and C elements obtained from the XPS spectra. The O/C ratios for the as-received GMC and as-received PMC samples were 0.004 and 0.001, respectively. These results indicate that both as-received nanomaterials present a low percentage of oxygen functionalities on their surfaces. After the treatment with H_2_SO_4_/HNO_3_, the O/C ratio increased to 0.045 and 0.218 for the GMCac and PMCac samples, respectively. This increase in the O/C atomic ratio indicates that the acid treatment has effectively introduced oxygen functional groups on the surface of both materials. The higher increase in the oxygen functionalities content of the PMC sample after the acid treatment (PMCac; 17.90%) than that of the GMC sample (GMCac; 4.39%) indicates that the PMC sample is more easily oxidized than the GMC sample, probably due to its larger number of surface defects.

The XPS C 1s high resolution spectra for the GMC, GMCac, PMC, and PMCac nanomaterials are shown in Figure 3B. Six main peaks were derived from curve fitting. The peaks obtained at 284.4 eV, 285.4 eV, 286.7 eV, 288.5 eV, and 290.1 eV are attributed to C-C, C-OH (phenol or alcohol), C = O (carbonyl or quinone groups), O-C = O (carboxyl, lactone, or ester groups), and carbonate groups, respectively (Chiang and Lee, 2009). The percentages of carbon and oxygen functionalities obtained from the deconvolution of the XPS spectra in the C 1s region is presented in Table 2. It is known that the oxidation reaction of carbon nanostructures occurs in two steps. The first step consists of the oxidant attack on the graphene structure by electrophilic reactions to generate active sites. The actives sites are sites on the nanoparticle surface where chemical functionalities are introduced, for instance C–OH groups (Santangelo et al., 2012). In fact, the C-OH groups are the oxygen functional groups that are present in higher percentage on the surface of all the carbon samples. The increase of the atomic % of C-OH groups observed for the PMC sample after the acid treatment (from 9.97 atomic% in the PMC to 16.74 atomic% in the PMCac) indicates that a significant amount of active sites were created on its surfaces during the oxidation process (Chiang and Lee, 2009). Meanwhile, only a small increase in the number of C-OH groups was observed after the acid treatment for the GMC sample. Thus, the generated surface defects on the GMC sample by the acid treatment is lower than that of the PMC sample, probably due to its higher graphitization index compared to that of the PMC sample. The second oxidation step consists of a process where the active sites generated in the first step are further oxidized and the aromatic rings are opened (Santangelo et al., 2012). During this process, some of the C-OH groups generated in the first oxidation step are consumed to produce O-C = O. In fact, the surface concentration of O-C = O functional group increases in both samples after the acid treatment, but in different proportions. The atomic percentage of O-C = O increases by 176.6% in the case of the PMC sample but only by 37.5% in the case of the GMC sample, which is in agreement with the fact that carbon nanomaterials with the lower graphitization index are easier to oxidize (Bi et al., 2008).

**Table 2:** Atomic percentages of the oxygen functional groups on the surface of the carbon nanoparticles obtained by XPS.

**Sample**	**Atomic %**					
	**C-OH**	**C=O**	**O-C=O**	**Carbonate**	**C**	**O**
GMC	12.21	8.12	2.40	3.40	99.60	0.40
GMCac	12.31	7.58	3.33	3.75	95.61	4.39
PMC	9.97	8.27	3.84	4.44	99.90	0.10
PMCac	16.74	11.99	10.62	4.47	82.10	17.90

**Figure 4:**
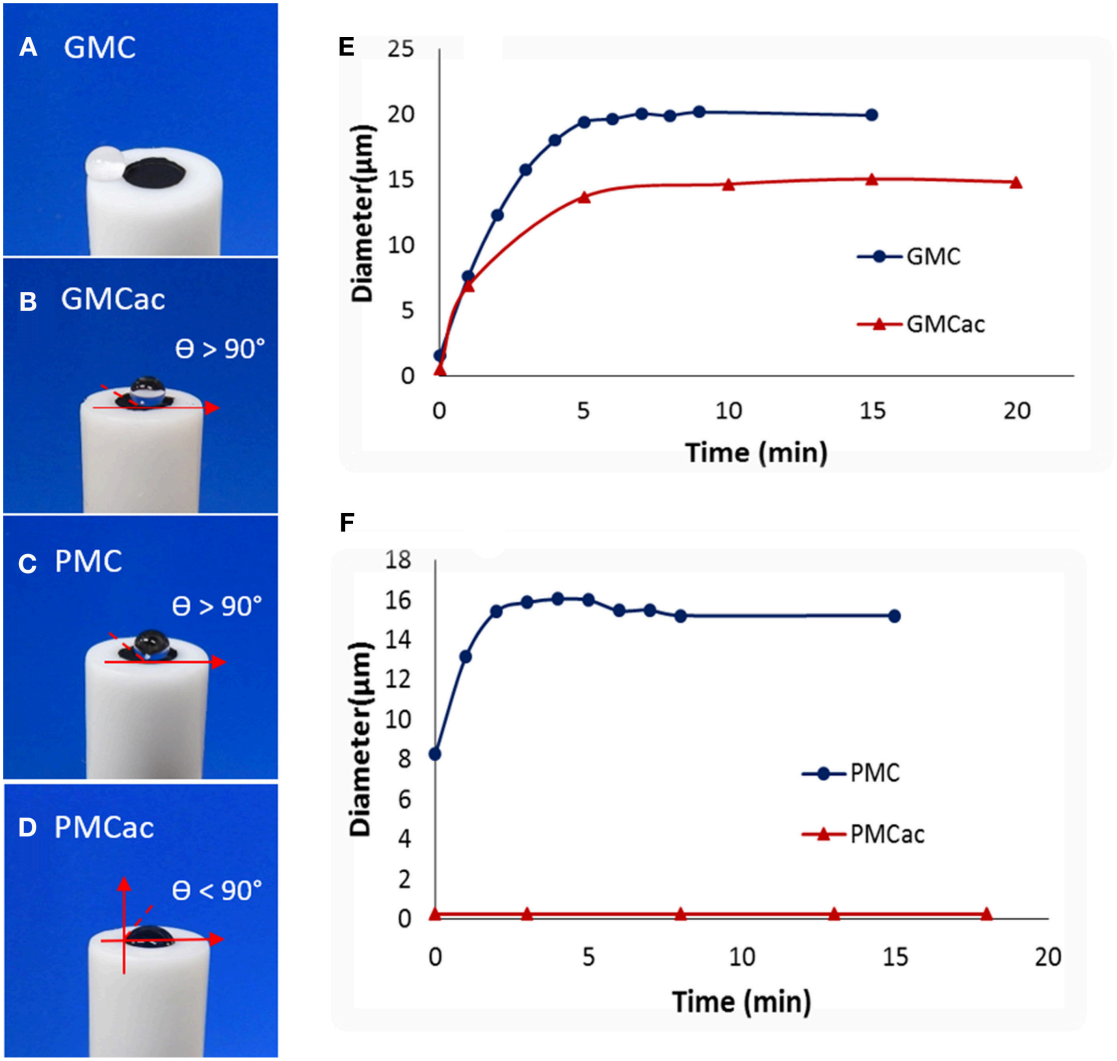
Images of a drop of water (20 μm) placed on films of **(A)** GMC, **(B)** GMCac, **(C)** PMC, and **(D)** PMCac. Dynamic light scattering measurements for the aggregation of **(E)** GMC/GMCac and **(F)** PMC/PMCac as a function of time.

Figure 4A shows that GMC presents a super-hydrophobic surface, where a drop of water jumps away from the carbon nanomaterial due to their lack of affinity. The acid treatment of this material increases its affinity for water, but the surface is still hydrophobic since a drop of water deposited on its surface has a contact angle higher than 90° (shown as θ > 90° in Figure 4B). Figure 4C shows that the as-received PMC is also a hydrophobic material. However, it has lower hydrophobicity than the as-received GMC (Figure 4A). This is in an agreement with the fact that the hydrophobicity of carbon materials decreases as the degree of graphitization decreases (Mattia et al., 2006). In contrast, the PMCac nanomaterial is hydrophilic, which shows a contact angle of <90° (Figure 4D). A high affinity of the PMCac nanomaterial for water is related to the large percentage of oxygen functionalities on its surface after the acid treatment.

The DLS measurements presented in Figure 4E indicate that the GMC and GMCac nanomaterials aggregate very quickly and form the aggregates with a diameter around 21 μm and 14 μm, respectively. The high aggregation rate for the GMC nanomaterial probably results from a combination of its high hydrophobic forces and low electrostatic repulsion among the individual GMC nanoparticles. The presence of oxygen functionalities on the surface of the GMCac nanomaterial would lead to a decrease of the hydrophobic forces and an increase of the electrostatic repulsions, producing a decrease in its aggregation rate. Meanwhile, the DLS measurements of the PMCac nanomaterial (Figure 4F) indicate its low tendency to form the carbon aggregates, which is probably due to “hydrophilic repulsions” between the individual PMCac nanoparticles. It has been reported that water molecules can adhere to hydrophilic particles (solvation phenomena) with enough energy to create a layer on its surface that prevents the nanoparticles from aggregating with each other (Nel et al., 2009).

### Electrochemical Characterization of GMC and PMC

**Figure 5:**
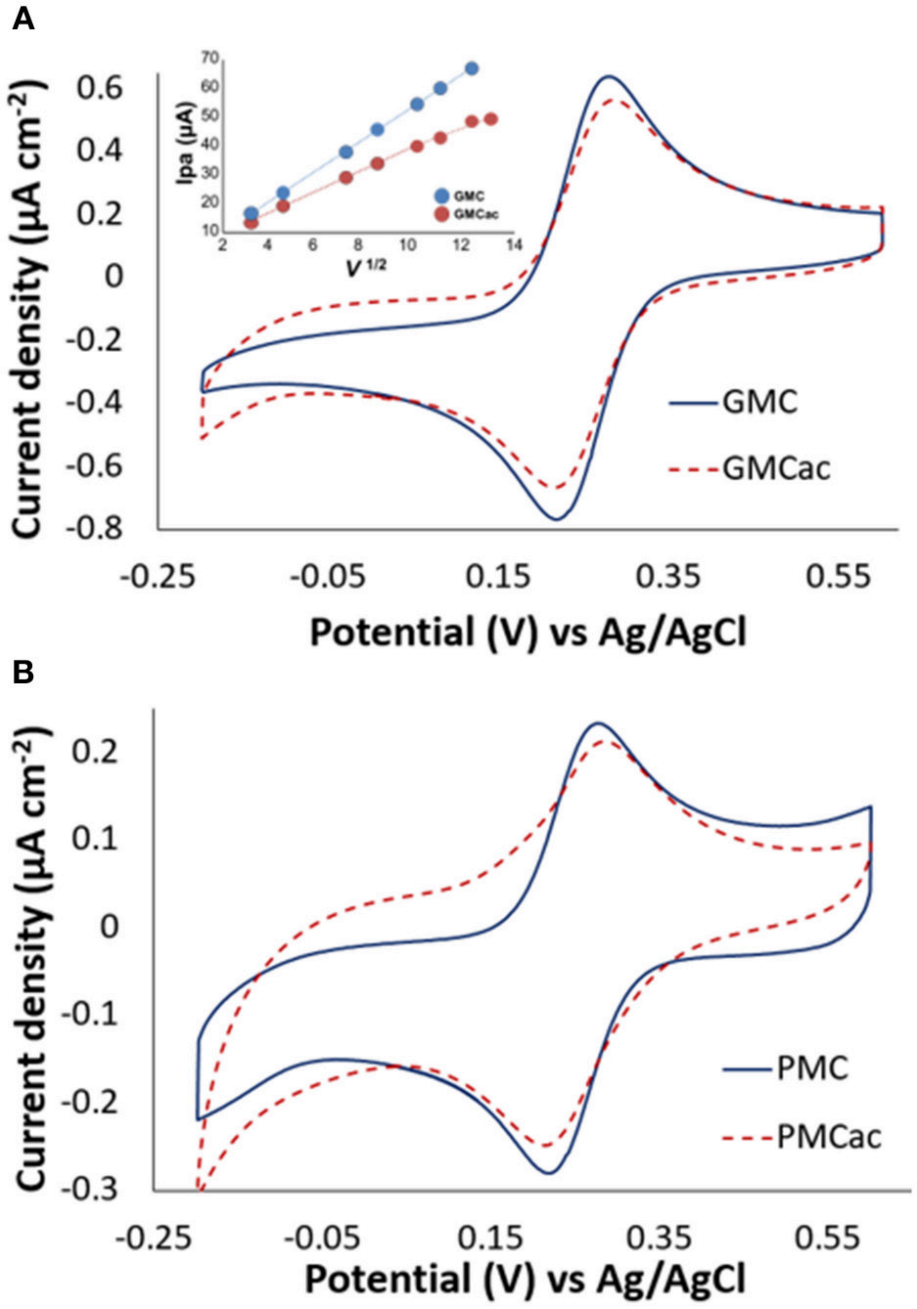
Cyclic voltammograms of **(A)** GMC/GMCac and **(B)** PMC/PMCac obtained in 1 mM of K_3_[Fe(CN)_6_] at 10 mV s^−1^. The inset in **A** shows the plot of *i*_*pa*_ *vs. v*^1/2^ *for GMC and GMCac*.

Figure 5 presents the CV plots for the GMC, GMCac, PMC, and PMCac nanomaterials obtained in the presence of 1 mM of potassium ferricyanide in PB (100 mM, pH 7.0) at the scan rate of 10 mV s^−1^. The CVs of the GMC and PMC show well-defined redox peaks at E_pc_ = +0.22 V (cathodic peak) and E_pa_ = +0.27 V (anodic peak). The separation between the peaks (Δ*E*_*p*_ = *E*_*pa*_ − *E*_*pc*_) in both cases is around 50 mV and the calculated formal potential was E° = +0.25 V vs. Ag/AgCl. This value is close to the formal potential calculated for the [Fe(CN)_6_]^3−/4−^ redox reaction for a glassy carbon electrode (E° = +0.24 V vs. Ag/AgCl), indicating the high electron transfer rate.

The effect of the acid treatment on the electrical conductivity of the carbon nanomaterials is also presented in Figure 5. The GMCac nanomaterial presents a 16.80% higher value of Δ*E*_*p*_ than that of the GMC nanomaterial (Figure 5A), while the PMCac nanomaterial presents a 27.20 % higher value of Δ*E*_*p*_ than that of the PMC nanomaterial at 10 mV s^−1^ (Figure 5B). This result suggests that the electron transfer capabilities of both the GMC and PMC samples have been negatively affected by the acid treatment, which may be due to the damage of their graphitic structures produced by its chemical oxidation in the H_2_SO_4_/HNO_3_ solution. The CV tests were also conducted at different scan rates. Based on these CV data, a plot of i_pa_ vs. v^1/2^ for the GMC and GMCac nanomaterials is constructed as shown in Figure 5A. The GMC sample presents a linear behavior with a slope of 5.53 μA mV^−1/2^ s ^½^ for the entire scan range. Therefore, the GMC sample is able to efficiently transfer electrons (i.e., reversible electron transfer) with the high electron transfer rates. Meanwhile, the GMCac sample presents a linear behavior with a slope of 3.86 μA mV^−1/2^ s ^1/2^, but only till 100 mV s^−1^, indicating that the redox reaction deviates from the behavior proposed by the Randles-Sevcik equation at the high scan rates. This suggests that the GMCac sample experiences an irreversible electron transfer at high scan rates, confirming that the GMCac sample is less electrically conductive than the GMC sample.

The peak-to-peak potentials (Δ*E*_*p*_) for the GMC and PMC samples at 100 mV-s are a) GMC: Δ*E*_*p*_ = 59.62*mV* and b) PMC: Δ*E*_*p*_ = 63.20 *mV*. Since higher values of Δ*E*_*p*_ are associated with lower electron transfer rates, these results indicate that the electron transfer rate of the PMC sample is smaller than that of the GMC sample. This difference is more important when the scan rate is increased (GMC: Δ*E*_*p*_ = 64.9 *mV*; PMC: Δ*E*_*p*_ = 75.25 *mV* at 200 mV-s). The presence of an ordered graphitic layer for the GMC sample is responsible for its enhanced electrical conductivity due to the localization of the π electrons (Portet et al., 2007; Lu et al., 2009). In summary, in terms of the electrical conductivity, the GMC nanomaterial is the best selection for making the high performance GOx-nanocomposite anode material.

### Morphology and Enzyme Loading of the GOx-Nanocomposites

**Figure 6:**
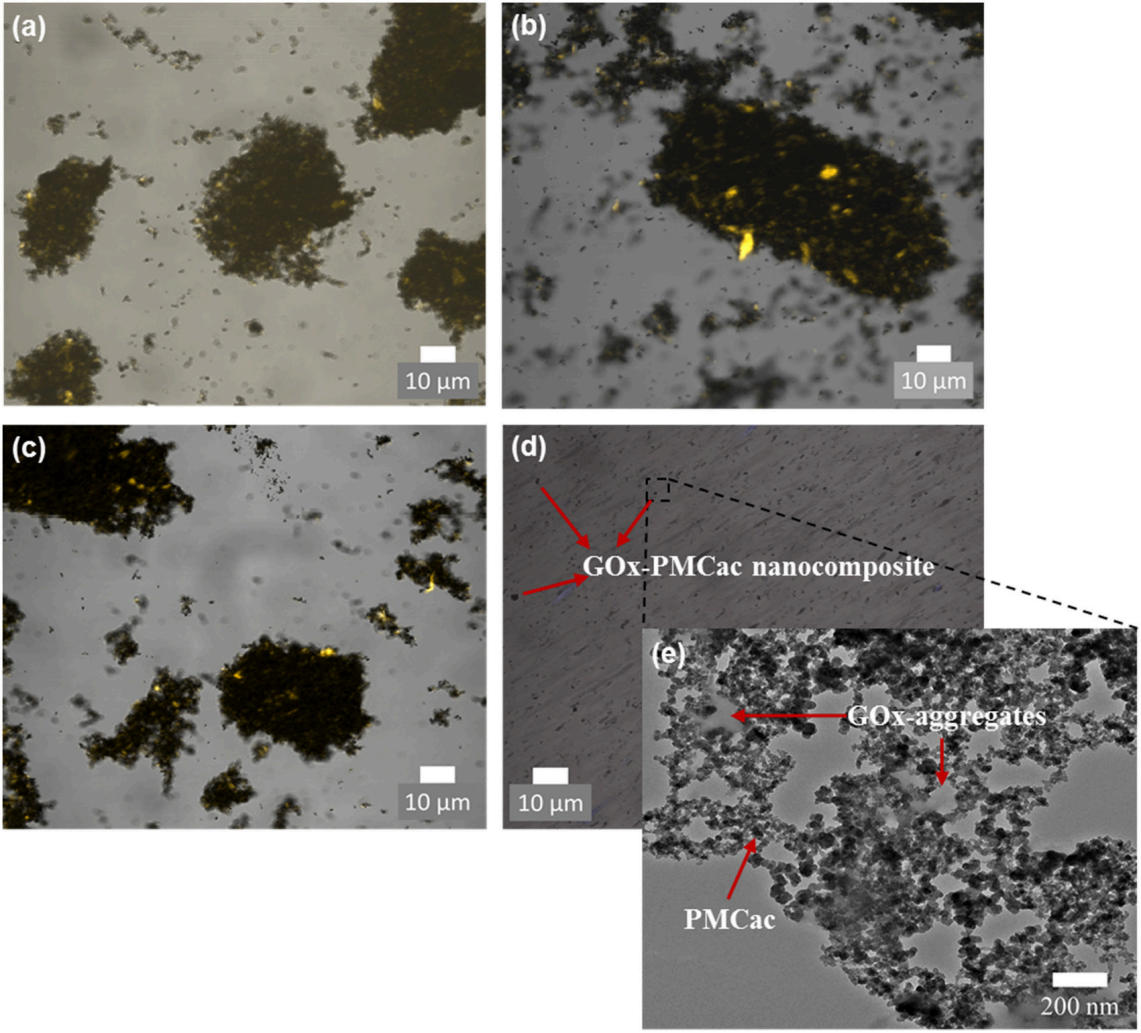
Confocal images of **(a)** GOx-GMC, **(b)** GOx-GMCac, **(c)** GOx-PMC, and **(d)** GOx-PMCac nanocomposites; and **(e)** TEM image of the GOx-PMCac nanocomposites.

Confocal microscopy was used to visualize the GOx-nanocomposites at higher magnifications (Figure 6). The locations of GOx aggregates in the confocal images were identified as bright spots due to the fluorescent property of the enzymes, while the carbon nanomaterials appear as black spots. Figures 6a–c show that the GOx-nanocomposites produced with the GMC, GMCac, and PMC samples present large enzyme aggregates. Thus, the physically entrapped enzyme aggregates are in a close contact with a large network of carbon nanomaterials. However, the GOx-PMCac nanocomposites are highly dispersed within the PB solution as shown in Figure 6d. This is in an agreement with the low aggregation tendency of PMCac observed in the DLS measurements. TEM tests were conducted in order to obtain a more detailed visualization of the small GOx-PMCac nanocomposites. The TEM image presented in Figure 6e shows the presence of GOx-aggregates within the PMCac network. This image suggests that the PMCac nanomaterial is able to entrap the smaller enzyme aggregates along with a much less compact carbon network structure compared to that of the GMC, GMCac, and PMC nanomaterials.

Our elemental analyses results showed that the nanocomposites contain 0.54 ± 0.05, 0.47 ± 0.01, 0.55 ± 0.11, and 0.45 ± 0.07 mg enzyme/mg of mesoporous carbon for the GOx-GMCac, GOx-GMC, GOx-PMCac, and GOx-PMC samples, respectively. These enzyme loading results suggest that the carbon nanomaterials are able to entrap similar amounts of enzymes per amount of carbon nanomaterials. Thus, any differences observed in their electrochemical performances cannot be attributed to differences in the enzyme loading but to the differences of carbon nanoparticles' physical properties.

### Electrochemical Behavior of GOx-Nanocomposites

**Figure 7:**
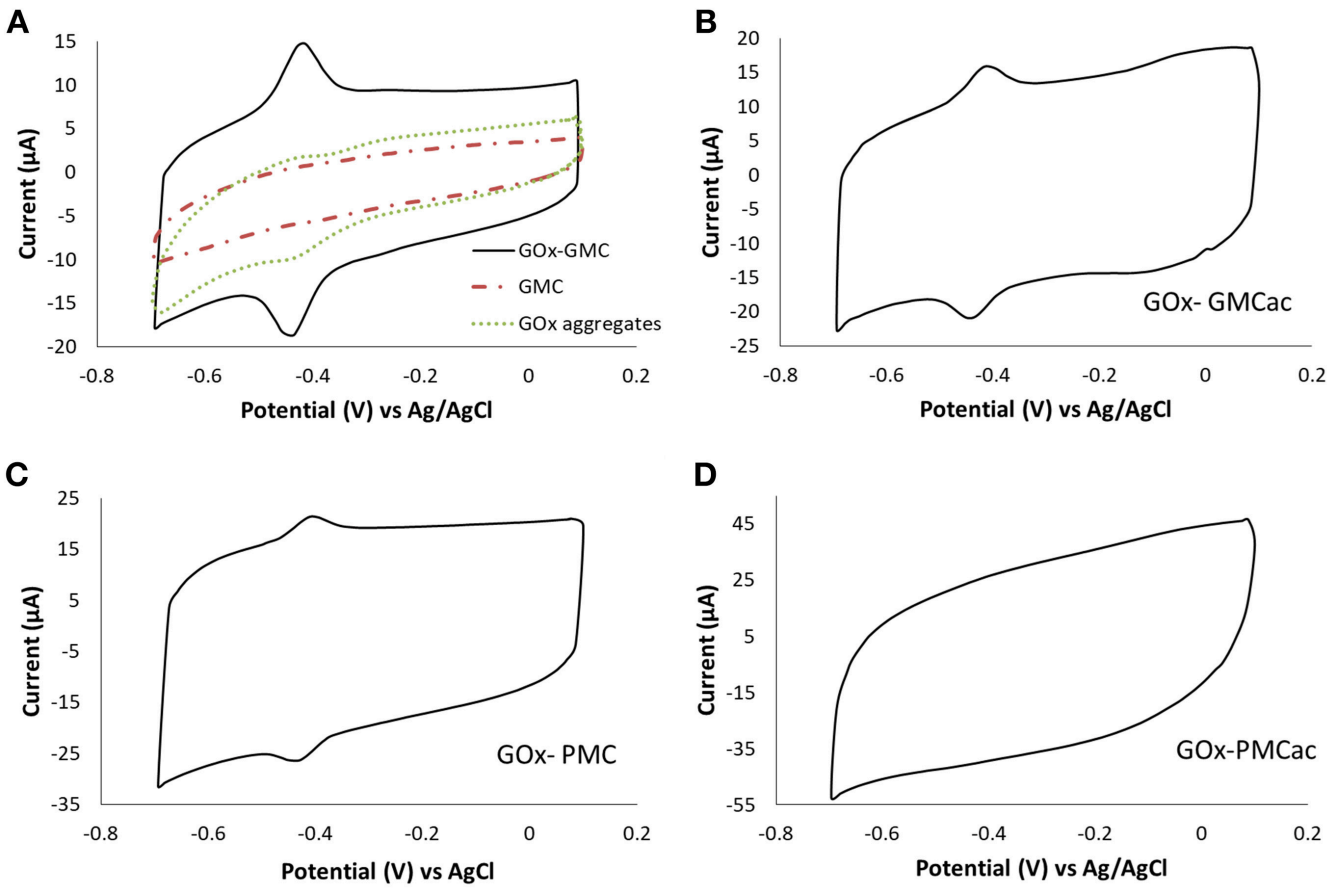
Cyclic voltammograms of **(A)** GOx-GMC, **(B)** GOx-GMCac, **(C)** GOx-PMC, and **(D)** GOx-PMCac nanocomposites. They are obtained in a N_2_ saturated PB electrolyte at a scan rate of 100 mV s^−1^.

The electrochemical performances of the GOx-nanocomposites were studied using CV tests. Figure 7 shows the CV plots obtained for the GOx-GMC, GOx-GMCac, GOx-PMC, and GOx-PMCac samples at 100 mV s^−1^ under the N_2_-saturated PB condition. Figure 7A presents the CV plots for the GOx-GMC sample, while the GOx aggregates and the GMC nanomaterial are shown as control. The CV plot for the GMC nanomaterial is featureless, indicating that the carbon nanoparticle by itself is not able to produce any electrochemical responses under the N_2_-saturated PB condition. The CV plot corresponding to the GOx aggregates shows a small redox peak. Meanwhile, the CV test for the GOx-GMC (Figure 7A) shows two well-defined redox peaks: an anodic peak at −0.437 V and a cathodic peak located at −0.419 V. The fact that an improved electrochemical activity is observed for the GOx-GMC sample when compared with the GOx aggregates and the GMC nanomaterial, suggest that the compact carbon network of the GOx-GMC sample provides the enhanced electron transfer process.

The formal potential (*E*_*o*_') calculated as $E_{0}^{\prime }=\frac{E_{pa}+\;E_{pc}}{2}$ was −0.429 V vs. Ag/AgCl, which is close to the formal potential of FAD/FADH_2_ (Guiseppi-Elie and Baughman, 2002; Liu et al., 2007; Vogt et al., 2014; Wilson, 2016). The origin of this peak is still not clear (Luong et al., 2016; Milton and Minteer, 2017). It is currently accepted that the direct electron transfer is very difficult to occur for GOx-based enzyme electrodes because the active site of GOx is buried inside its protein structure and the surface of GOx is covered by non-conductive glycosylation layer (Wilson, 2016). This peak has been ascribed to adsorbed free FAD (flavin adenine dinucleotide) on the carbon nanomaterials or the presence of impurities in the commercial GOx (Vogt et al., 2014; Wilson, 2016). Some authors also suggested that small traces of O_2_ in the solution can also promote pseudo-direct electron transfer (pseudo-DET) (Milton and Minteer, 2017). Determining the electron transfer mechanism between the active site of GOx and the electrode surface requires further study and is beyond the scope of this paper. The CV curves obtained for the GOx-GMCac and GOx-PMC nanocomposites also exhibit two redox peaks at around −0.429 V vs. Ag/AgCl (Figures 7B,C). Conversely, the GOx-PMCac sample shows a featureless CV plot indicating its low electrochemical activity.

According to Figure 7, the redox peaks of the GOx-GMC sample show higher current peak intensities than that of the GOx-GMCac and GOx-PMC samples (Ipa = 6.63 μA for the GOx-GMC vs. Ipa = 4.23 μA for the GOx-GMCac and 3.13 μA for the GOx-PMC). This may result from the enhanced electrical conductivity of the GMC sample, which arises from its high graphitization index and low concentration of oxygen functionalities on its surface (Datsyuk et al., 2008). The peak-to-peak potentials (Δ*E*_*p*_) were 19.8, 27.06, and 39.72 mV for the GOx-GMC, GOx-GMCac, and GOx-PMC samples, respectively. It is known that this parameter is directly related to the electron transfer rate constant (k_s_) (i.e., the decreased separation between the redox peaks indicates the faster electron transfer rate during the charge transfer reaction).

The electron transfer rate constant (k_s_) for each sample was also determined by using the Laviron method for ΔE_p_ is <200*/n* mV (*n* is the number of electrons transferred during the reaction) and the transfer coefficient value varies between 0.3 and 0.7 (Laviron, 1979). The k_s_ for the GOx-GMC, GOx-GMCac, and GOx-PMC nanocomposites were 6.63, 4.57, and 2.74 s^−1^, respectively. Therefore, the GOx-GMC nanocomposite displayed the highest electron transfer rate, confirming that the as-received GMC is the best electron transfer promoting carbon nanomaterial. The value of k_s_ cannot be estimated for the GOx-PMCac sample since it does not show any distinctive redox peak. This result indicates that the PMCac sample doesn't facilitate the efficient electron transfer process in the system due to its low conductivity and lack of continuous carbon network.

The mechanism governing the electron transfer process in the GOx-based enzyme electrodes has been subjected to intense research with various interpretations (Vogt et al., 2014; Luong et al., 2016). This paper doesn't aim to determine the electron transfer mechanism in our GOx-nanocomposite materials. Instead, we intend to show the effect of employing different mesoporous carbons with various surface and bulk properties on the electrochemical performances of the GOx-nanocomposites.

**Figure 8:**
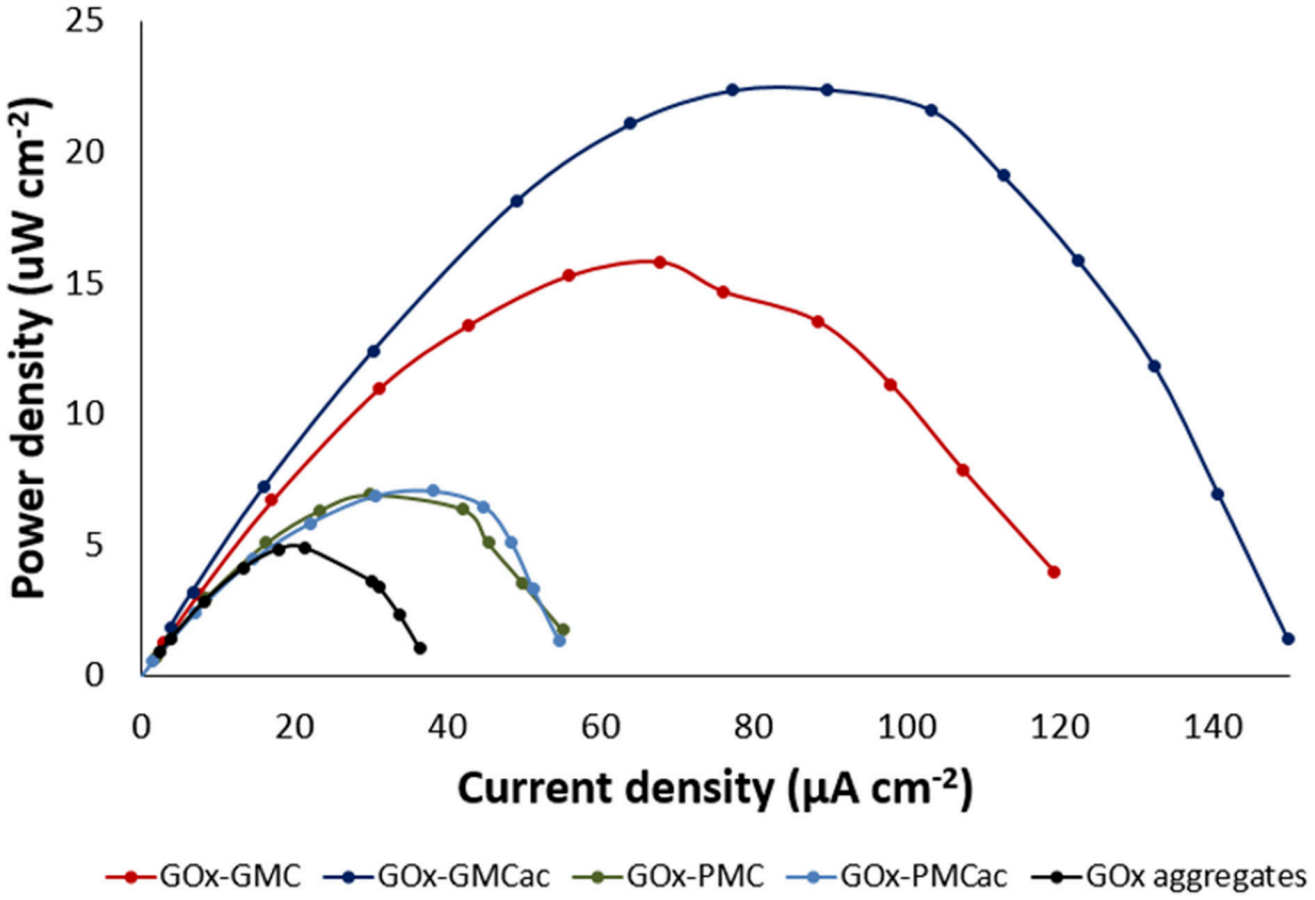
Biofuel cell power densities, measured in 10 mM glucose solution at room temperature for GOx-GMC, GOx-GMCac, GOx-PMC, GOx-PMCac nanocomposites and GOx-aggregates anodes.

Figure 8 shows the power density of the GOx-nanocomposites obtained in the BFC, using 10 mM glucose solution as the fuel. The values of maximum power density for the BFCs with the GOx-GMCac, GOx-GMC, GOx-PMC and GOx-PMCac bioanodes are 22.40, 15.80, 7.06, and 6.89 μW/cm^2^, respectively. Because the enzyme loadings in all cases are similar, any differences observed in their maximum power density would be attributed to differences in carbon nanoparticles' properties.

The BFC with the GOx-GMC bioanode produces 2.2 times the power density of the BFC with the GOx-PMC bioanode (15.80 vs. 7.06 μW/cm^2^). Furthermore, the BFC with the GOx-GMCac bioanode produces 3.2 times the power density of the BFC with the GOx-PMCac bioanode (22.40 vs. 6.89 μW/cm^2^). As described in the earlier section, the GMC sample offers a more ordered carbon bulk structure and higher electrical conductivity than that of the PMC sample. Hence, our BFC results can be attributed to the higher electrical conductivity offered by the GMC nanomaterial than the PMC nanomaterial. However, if the electrical conductivity of nanomaterials is the only key parameter that determines the power density of the BFC, the BFC with the GOx-GMC bioanode should provide the highest power density because the GOx-GMC offers the highest electrochemical properties and highest electron transfer rate constant (k_s_) as shown in their CV tests (Figure 5). Nevertheless, according to Figure 8, the highest power density output was obtained from the BFC with the GOx-GMCac bioanode. This result suggests that the power density of the BFC does not solely depended on the electrical conductivity of the nanomaterials.

To understand this unexpected result, we need to consider the differences in the morphology of the carbon nanomaterial aggregates used. According to TEM images shown in Figure 6, the bright spots indicate the enzyme aggregates. It seems that the GOx-GMC sample offers a higher degree of carbon packing than that of the GOx-GMCac sample. Thus, a smaller number of bright spots (i.e., the enzyme aggregates) are exposed to the surface for the GOx-GMC sample than the GOx-GMCac sample. According to Figure 4, the GOx-GMC sample possesses the super hydrophobic surface property where the GOx-GMCac sample shows the decreased surface hydrophobicity due to the increased number of the surface functional group. Since the dispersion of the carbon nanomaterial in the aqueous media decreases as its surface hydrophobicity increases, the GOx-GMC sample with the higher surface hydrophobicity leads to the GOx nanocomposite material with the more compact carbon network structure than that of the GOx-GMCac sample. For the GOx-GMC sample, it seems that the carbon packing is too high where the carbon nanomaterials cover much of the enzyme aggregates at its surface and it would prevent the efficient mass transport of the fuel to the enzymes. Addition to the poor mass transport of the fuel over the surface of the GOx-GMC sample, it could also produce a very tight carbon network structure with low available internal void spaces for the efficient fuel transportation within the nanocomposite structure (Catalano et al., 2015). Consequently, the BFC with the GOx-GMCac bioanode produces the higher power density than that of the GOx-GMC bioanode where it offers both the high electrical conductivity and the efficient mass transport of the fuel.

In contrast to the GOx-GMC sample, the GOx-PMCac sample shows the most open carbon network structures with a greater number of enzyme aggregates that are exposed to the surface (Figure 6). Such structure would allow the high mass transport of the fuel, but it would lead to a very poor electrical conductivity because it is unable to form a continuous carbon network for the electrons to efficiently move through the system. Consequently, the BFC with the GOx-PMCac produces the one of the worst power density outputs as shown in Figure 8. This finding suggests that the electrochemical performance of the GOx-bioanodes is not only depended on the bulk properties (e.g., graphitization index and electrical conductivity) of the carbon nanomaterials, but it is also depended on the surface properties (e.g., concentration of surface functional group and degree of surface hydrophobicity) of the carbon nanomaterials to form the continuous carbon network structure.

## Conclusions

The electrochemical performances of four GOx-nanocomposites manufactured by using four different carbon nanomaterials as the supports were studied. Our findings indicate that the physical properties of carbon nanomaterials significantly affect the electrochemical performances of the GOx-nanocomposites produced by immobilizing GOx-aggregates within a network of these carbon nanomaterials. The GOx-GMC sample offers the most efficient electron transfer rate due to its highly ordered crystalline structure and compacted carbon network structure. On the other hand, the GOx-PMCac sample offers the least efficient electron transfer rate due to its turbostratic carbon structures with a low degree of bulk organization and its inability to form the continuous carbon network (i.e., its high dispersion nature) in the aqueous media. Consequently, the BFC with the GOx-GMC bioanode produces the higher power density output than that of the GOx-PMCac bioanode. However, the highest power density of the BFC can be obtained when both the high electrical conductivity and efficient mass transport of the fuel were achieved. To this regard, the GOx-GMC sample is not the best bioanode material because its TEM image suggests that its resulted carbon network is too tight and degree of carbon aggregate is too high for achieving the efficient mass transport of the fuel. When the GMC sample is acid treated to form the GMCac, additional surface defects were introduced decreasing both the surface hydrophobicity and the electron transport efficiency, while improving the mass transport of the fuel by reducing its tendency to form the tight carbon network. The BFC with the GOx-GMCac bioanode produced the highest maximum power density output of 22.40 μW/cm^2^, which is about 42 % higher than that of the GOx-GMC bioanode. This result suggests that the positive effect of the acid treatment for the GMC material (i.e., improving the mass transport of the fuel) outweighs its negative effect (i.e., decreasing the electrical conductivity). Therefore, the GOx-nanocomposites as the effective enzyme anodes for various electrochemical applications should achieve both the high electronical conductivity and efficient mass transport of the fuel by optimizing not only its bulk property (e.g., electrical conductivity) but also optimizing its surface property (e.g., surface hydrophobicity).

## Author Contributions

TG-P gave significant efforts to obtain Figures 1, 2, 4–7. SH and LS gave significant efforts to obtain Figure 3. CH gave significant efforts to obtain Figure 8. YW gave significant efforts to develop the procedure for creating the protein aggregates for this manuscript. JK supervised our efforts to create the enzyme anode using the protein-nanocomposites. SuH supervised our efforts to conduct the physical and electrochemical property measurements of protein-nanocomposite-based enzyme anode and its biofuel cells.

### Conflict of Interest Statement

The authors declare that the research was conducted in the absence of any commercial or financial relationships that could be construed as a potential conflict of interest.
